# Editorial: Advances in skin immunology

**DOI:** 10.3389/fimmu.2025.1755100

**Published:** 2026-01-06

**Authors:** Olga Simionescu

**Affiliations:** 1 Carol Davila University of Medicine and Pharmacy, Bucharest, Romania; 2 Department of Dermatology, Colentina Clinical Hospital, Bucharest, Romania

**Keywords:** autoimmune disorders, cytokines, immune therapy, interleukins, skin immunology, immune circuits

## 1 Introduction

The human skin does not fit the classic definition of a secondary lymphoid organ as it lacks lymphatic follicles, organised follicular dendritic cells and an architectural compartment containing T or B cells. However, functionally, it behaves like a secondary lymphoid organ and “immune orchestrator”. It is equipped with stable memory cells (1) and is the site where adaptive responses begin (2). Furthermore, in inflammatory and/or autoimmune conditions such as psoriasis, eczema and chronic lupus erythematosus (LE), the local production of antibodies and the aggregation of T and B cells can be equated with tertiary lymphoid structures (3).

As guest editor of the special series “Advances in Skin Immunology ‘, I analysed papers that share a dermatological component, some of which report rare cases.

But the guiding thread of the edition the role of skin immunity as an ‘orchestrator’ in inflammation, metabolism and targeted therapies (4).

## 2 The skin’s immune and metabolic balance

*Patients with vitiligo* can benefit from quitting tobacco and alcohol consumption, as well as taking vitamins E and B12, and the minerals copper (Cu) and zinc (Zn). (Liang et al.). The link between melanocyte survival and modulation of the immune response *via* vitamin D receptor (VDR) agonists suggests that these may be potential candidates for treating vitiligo (5).

Not only is the skin a self-regenerating organ, it also performs its own tissue repair in the event of injury. Fibrosis occurs as a defence mechanism *via* the involvement of macrophages in hypertrophic scarring. Type 1 macrophages intervene in early inflammation, secreting proinflammatory cytokines (Shen et al.), while type 2 macrophages appear at a later stage, during tissue repair and fibrosis consolidation.

A meta-analysis of 116 studies examining autoimmune and metabolic comorbidities in *primary scarring alopecia* (Yongpisarn et al.) found significant associations with systemic lupus erythematosus (SLE), hypothyroidism, metabolic diseases and dermatological conditions.

## 3 Rare phenotypes with disrupted immune circuits are expressed in the molecular architecture of the skin

Although mucosal membrane involvement in patients with *Linear immunoglobulin A (IgA) bullous dermatosis (LABD)* is rare, Kowalewski and Wozniak demonstrate that severe scarring of the oesophagus and conjunctiva can occur in the presence of circulating IgG and IgA antibodies to the LAD-1 antigen. Clinicians should be aware that ‘Linear IgA deposits in the Basement Membrane Zone (BMZ) detected by Direct ImmunoFluorescence (DIF) are not pathognomonic for LABD (Kowalewski and Wozniak).

Disrupted immune circuits are also found in other bullous diseases, such as immune pemphigus (6).

All interleukin 17 (IL-17) isoforms signal through IL17 receptor (IL-17R), the common subunit of all heteroreceptors of which IL-17RA is one (7). The presence of psoriasis and chronic mucocutaneous candidiasis in a five-year-old child born to consanguineous parents has been linked to a biallelic variant in the IL17 receptor A gene (Kadhi et al.).

Another important association is that between neonatal lupus, which is uncommon (8) and atopic dermatitis (AD) (Sun et al.). While the primary manifestations of neonatal lupus erythematosus (LE) are cutaneous and cardiac, due to the transplacental passage of maternal antibodies (anti-Ro/SSA or anti-La/SSB), it may be associated with AD, in a relationship that is unclear with perinatal factors. A retrospective cohort study reports that oral administration of probiotics in children with neonatal LE reduces the risk of AD (Sun et al.). This can be viewed as an example of maternal-foetal immune cross-talk, whereby disease is generated if maternal tolerance towards the foetus is disrupted.

*Molecular analysis of checkpoint inhibitors* associated with toxicities, such as polymorphic erythema in a patient with isolated brain metastases (melanoma) and erythema nodosum (Sun et al.), demonstrates how blocking immune regulation can reveal hidden inflammatory pathways. Immune checkpoint inhibitors (ICIs) block the interaction between programmed death-1 (PD-1) and cytotoxic T lymphocyte antigen-4 (CTLA-4) and their respective ligands, which are found on the surfaces of antigen-presenting cells (APCs) or tumour cells (PD-L1/2 and CD80/86) (9). On the other hand, erythema nodosum is not an autoimmune disease, but rather a reaction to superantigens (10).

This analysis shows that adverse reactions to treatment with checkpoint inhibitors differ from the tumour immune response due to the unique immune landscape of Erithema nodosum *like* immune-related adverse events (EN-like irAEs) (Sun et al.).

Another example of immunological mechanisms in rare cases is that of an 87-year-old female uremic patient undergoing peritoneal dialysis (Zhang et al.) who developed *psychogenic purpura*. This is a rare autoimmune vasculopathy also known as Gardner–Diamond syndrome or autoerythrocyte sensitisation syndrome (11). The pathology of purpura induced by psychological stress is not fully understood.

## 4 A “tailor-made” approach to modern immunological therapy, with a focus on the benefit-risk ratio

In recent years, *modern immunotherapy* has effectively transformed the approach to treating patients with oncological and inflammatory diseases (4). Depending on the geography and reimbursement system of each country, some molecules are included in national protocols, while others are not, as treatments are expensive. These neuro-immune signalling molecules require a personalised approach.

Thus, *treatment with anti-IL36 has been reported for generalized pustular psoriasis (GPP)* associated with Hallopeau’s acrodermatitis after conventional therapies have failed (Hou et al.). Adverse reactions were not significant. IL-36 belongs to the IL-1 cytokine superfamily and is a cytokine whose signalling imbalance favours proinflammatory activity as a main driver of the pathogenesis of GPP (12). This explains the rapid impact of IL-36 inhibitors and why Spesolimab has been classified as a ‘first-in-class medication’ for treating acute GPP flares.

Vulgaris psoriasis, another form of psoriasis, was aggravated in a 61-year-old cancer patient who was treated with sintilimab for pulmonary metastases secondary to colon cancer (Ke et al.). In this case, guselkumab (also an IL-23 inhibitor) was introduced and there was a significant improvement in psoriasis symptoms, while the pulmonary condition remained stable during follow-up after standard cancer therapy was completed. *Combining two biological therapies in a frail cancer patient* is a modern approach based on interfering with systemic cancer-dermatology immune processes.

Proinflammatory cytokine profiles are distinct in patients with *nodular prurigo*, some of whom may have atopic dermatitis (Lee et al.). *Transcriptomic analysis* of these patients demonstrates that cytokine-mediated pathways play an important role in the pathogenesis of AD, highlighting the importance of identifying molecular targets. The pathogenesis of prurigo nodularis, which is still incompletely understood, involves cross talk between sensory nerve fibres, immune cells and the epidermis (13).

*Iatrogenic vitiligo-like depigmentation (VDL)* has been reported in cancer patients undergoing immunotherapy, with a notably high incidence in cases of melanoma and chronic myeloid leukaemia. In melanoma patients treated with pembrolizumab, the incidence of VDL was 2–25% due to an enhanced immune response destroying melanocytes via shared antigens (Wang et al.). In patients with chronic myeloid leukaemia treated with imatinib, vitiligo-like depigmentation occurs through the inhibition of the x-Kit/SCF signalling pathway, with an incidence of 40.9% (Wang et al.). The incidence of VDL was insignificant in cases of autoimmune inflammation (psoriasis, atopic dermatitis, inflammatory bowel disease, multiple sclerosis and rheumatoid arthritis) treated with IL-17, IL-23, IL-4R or TNF-α inhibitors (Wang et al.).

*Non-pharmacological neuroimmune modulation* may be considered for *chronic* sp*ontaneous urticaria* (CSU) (Wei et al.). A meta-analysis of 1,867 patients and 22 randomised controlled trials (RCTs) by experts in traditional Chinese medicine showed that acupuncture is clinically effective and safe. It reduces the number and size of wheals, shortens the duration of flare-ups and reduces serum IgE, IFN-γ and IL-4 levels.

## 5 Discussions

‘Advances in Skin Immunology’ is a special series of papers exploring the role of skin immunity in specific inflammatory and neoplastic conditions. Present-day and future medicine (2025+) is evidence-based and precise. Although the skin is not a peripheral lymphoid organ, its behaviour is similar, with multiple integrative and dynamic levels.

A precise characterisation of skin immunity enables new immunological treatments to be applied, which require clinical and molecular integration. For this reason, it is imperative to thoroughly analyse the relationship between metabolism and skin immunity and the immune circuits disrupted in rare phenotypes. Last but not least, the benefit-risk ratio of modern immunological therapy must be considered, as each patient is unique.
